# Neolignans from *Selaginella moellendorffii*

**DOI:** 10.1007/s13659-016-0095-5

**Published:** 2016-04-07

**Authors:** Jing-Xian Zhuo, Yue-Hu Wang, Xing-Li Su, Ren-Qiang Mei, Jun Yang, Yi Kong, Chun-Lin Long

**Affiliations:** 1 Key Laboratory of Economic Plants and Biotechnology, and Yunnan Key Laboratory for Wild Plant Resources, Kunming Institute of Botany, Chinese Academy of Sciences, Kunming, 650201 China; 2 School of Life Science & Technology, China Pharmaceutical University, Nanjing, 210009 China; 3 College of Life and Environmental Sciences, Minzu University of China, Beijing, 100081 China

**Keywords:** Selaginellaceae, *Selaginella moellendorffii*, Lignans, Antiplatelet

## Abstract

**Abstract:**

Two new neolignans selaginellol (**1**) and selaginellol 4′-*O*-β-d-glucopyranoside (**2**), together with seven known compounds (**3**–**9**), were isolated from the whole plant of *Selaginella moellendorffii*. The structures of the new isolates were determined through spectroscopic data analysis. Compounds **1**–**9**, as well as compounds **10**–**18** previously isolated from the species, were measured for the activity against platelet aggregation induced by ADP or collagen. Three neoligans (**8**, **11**, and **12**), one flavanone (**14**), and one alkaloid (**16**) showed inhibitory activity against ADP- or collagen-induced platelet aggregation as compared with tirofiban. The dihydrobenzofuran neolignans (**8**, **11**, and **12**) are more potent than the benzofuran neolignan (**13**) and other types of neolignans (**1**–**7**). Glucosidation of the dihydrobenzofuran neolignans (**11** and **12**) is helpful for the activity.

**Graphical Abstract:**

Two new neolignans selaginellol (**1**) and selaginellol 4′-*O*-β-d-glucopyranoside (**2**) were isolated from the whole plant of *Selaginella moellendorffii*. Several compounds from this plant showed the activity against platelet aggregation induced by ADP or collagen.
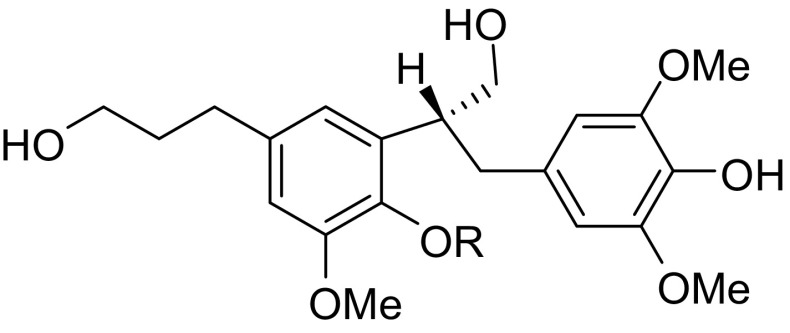

**Electronic supplementary material:**

The online version of this article (doi:10.1007/s13659-016-0095-5) contains supplementary material, which is available to authorized users.

## Introduction

**Fig. 1:**
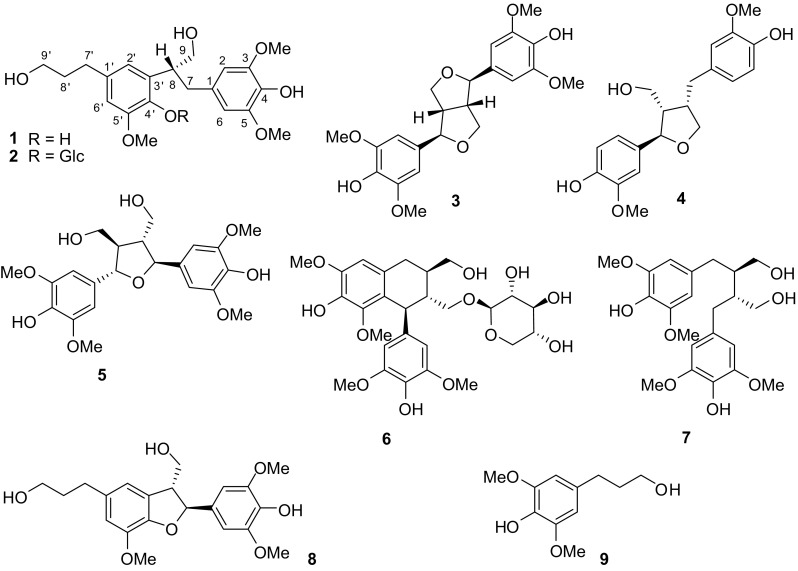
The chemical structures of **1**–**9** from *Selaginella moellendorffii*

The family Selaginellaceae Willk. includes the single genus *Selaginella* Beauv. *Selaginella* is a nearly worldwide genus of about 700 species, with 72 of them in China and more than 20 species used in traditional Chinese medicine [1, 2]. Several *Selaginella* species including *S. delicatula* (Desv. ex Poir.) Alston, *S. moellendorffii* Hieron., *S. nipponica* Franch. & Sav., *S. sanguinolenta* (L.) Spring, *S. stauntoniana* Spring, and *S. tamariscina* (P. Beauv.) Spring are used in promotion of blood circulation (Huoxue in Chinese) [1]. Traditional Chinese medicines with the functions of “Huoxue” and/or “Huayu” (removing blood stasis) are claimed to be useful in antiplatelet therapies and the treatment of thrombotic diseases [3, 4]. Previously, a pyrrolidinoindoline alkaloid selaginellic acid with antiplatelet activity was found from the whole plant of *S. moellendorffii* [5, 6]. This result prompted us to further investigate the plant which led to the isolation of nine compounds (**1**–**9**, Fig. 1) including two new neolignans (**1** and **2**). Compounds **1**–**9**, as well as those (**10**–**18**) previously isolated from the plant [5, 7, 8], were evaluated for antiplatelet activity. The structural elucidation of the new compounds and the bioassay results are reported.

## Results and Discussion

The HRESIMS analysis of selaginellol (**1**) gave an [M+Na]^+^ ion at *m/z* 415.1729 appropriate for a molecular formula of C_21_H_28_O_7_ requiring eight sites of unsaturation. The IR absorption signals revealed the presence of hydroxy (3428 cm^−1^) and aromatic (1614, 1518, 1496, and 1461 cm^−1^) groups. The ^1^H NMR data of **1** (Table 1) exhibited three methoxy groups [*δ*_H_ 3.82 (3H, s) and 3.70 (6H, s)], and two 1,2,3,5-tetrasubstituted benzene rings [*δ*_H_ 6.30 (2H, s); 6.63 (d, *J* = 1.6 Hz) and 6.47 (d, *J* = 1.6 Hz)]. The ^13^C NMR data of **1** (Table 1) showed the signals for three methoxy groups (*δ*_C_ 56.5 × 2 and 56.4), two phenyl rings, five methylenes including two oxygenated ones (*δ*_C_ 65.9, 62.2, 37.9, 35.8, and 32.8), and one methine (*δ*_C_ 45.4). According to above NMR signal characteristics [8], compound **1** might be a neolignan.

**Table 1:** ^1^H (600 MHz) and ^13^C (150 MHz) NMR data of **1** and **2** in CD_3_OD (*δ* in ppm, *J* in Hz)

Position	**1**		**2**	
	*δ*_H_	*δ*_C_	*δ*_H_	*δ*_C_
1		132.9 (C)		132.5 (C)
2,6	6.30 (s)	107.3 (CH)	6.28 (s)	107.0 (CH)
3,5		148.7 (C)		148.6 (C)
4		134.2 (C)		134.1 (C)
7	3.00 (dd, 13.4, 5.7) 2.88 (dd, 13.4, 9.4)	37.9 (CH_2_)	3.01 (dd, 14.0, 5.0) 2.71 (dd, 14.0, 10.0)	39.6 (CH_2_)
8	3.42 (m)	45.4 (CH)	3.96 (m)	42.7 (CH)
9	3.76 (m)	65.9 (CH_2_)	3.76 (dd, 10.6, 4.8) 3.67 (dd, 10.6, 7.9)	67.1 (CH_2_)
1′		133.7 (C)		140.4 (C)
2′	6.47 (d, 1.6)	122.0 (CH)	6.73 (d, 1.8)	120.2 (CH)
3′		129.3 (C)		138.5 (C)
4′		143.7 (C)		143.5 (C)
5′		148.7 (C)		153.2 (C)
6′	6.63 (d, 1.6)	110.6 (CH)	6.72 (d, 1.8)	111.6 (CH)
7′	2.53 (t, 7.5)	32.8 (CH_2_)	2.64 (t, 7.6)	33.1 (CH_2_)
8′	1.74 (m)	35.8 (CH_2_)	1.82 (m)	35.7 (CH_2_)
9′	3.51 (t, 6.4)	62.2 (CH_2_)	3.57 (t, 6.2)	62.2 (CH_2_)
1″			4.57 (d, 7.5)	105.6 (CH)
2″			3.44 (m)	75.9 (CH)
3″			3.39 (m)	77.8 (CH)
4″			3.38 (m)	71.1 (CH)
5″			3.11 (m)	78.0 (CH)
6″			3.79 (overlapped) 3.70 (overlapped)	62.4 (CH_2_)
3,5-OMe	3.70 (s)	56.5 (CH_3_)	3.70 (s)	56.5 (CH_3_)
5′-OMe	3.82 (s)	56.4 (CH_3_)	3.80 (s)	56.3 (CH_3_)

**Fig. 2:**
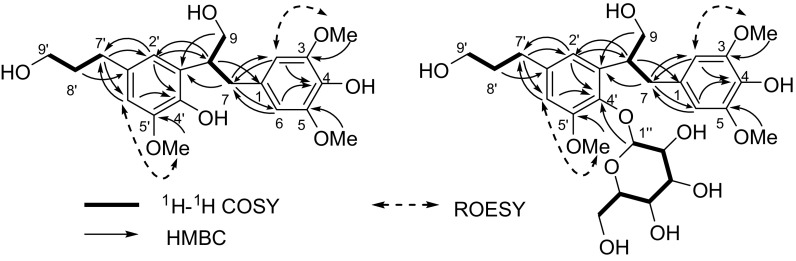
Key 2D NMR correlations of **1** and **2**

The ^1^H–^1^H COSY correlations (Fig. 2) exhibited two partial structures from C-7 to C-9 and C-7′ to C-9′. Based on the HMBC correlations (Fig. 2) from H-2 and H-6 to C-4, H_2_-7 to C-2 and C-6, H-8 to C-1, H_2_-8′ to C-1′, H_2_-7′ to C-2′ and C-6′, H-2′ and H-6′ to C-4′, 3-OMe to C-3, 5-OMe to C-5, and 5′-OMe to C-5′, two phenylpropanoid moieties, namely 4-(3-hydroxypropyl)-2,6-dimethoxyphenol and 4-(3-hydroxypropyl)-2-methoxyphenol, were confirmed. The two fragments were linked through C-8-C-3′ by the HMBC correlations from H_2_-7 and H_2_-9 to C-3′ as well as H-8 to C-2′ and H-2′ to C-8. Therefore, the relative configuration of **1** was elucidated as 3,5,5′-trimethoxy-8,3′-neoligna-4,4′,9,9′-tetraol. The absolute configuration of selaginellol (**1**) was elucidated as (8*R*)-3,5,5′-trimethoxy-8,3′-neoligna-4,4′,9,9′-tetraol by comparing its electronic circular dichroism (ECD) spectrum [Δ*ε* −0.12 (273)] with that of a known analogue secodihydrodehydrodiconiferyl alcohol tetraacetate [9].

According to the HREIMS ion at *m/z* 554.2365 [M]^+^ (calcd for C_27_H_38_O_12_, 554.2363), the molecular formula of compound **2** was determined as C_27_H_38_O_12_ with nine degrees of unsaturation. The IR absorption signals showed the presence of hydroxy (3425 cm^−1^) and aromatic (1614, 1518, and 1461 cm^−1^) groups. The NMR data (Table 1) of **2** were very similar to those of **1**, except that more signals for a β-glucopyranosyl moiety [*δ*_H_ 4.57 (d, *J* = 7.5 Hz); *δ*_C_ 105.6, 75.9, 77.8, 71.1, 78.0, and 62.4] were observed. As demonstrated in the ^1^H–^1^H COSY, HMBC and ROESY correlations (Fig. 2), compound **2** was determined to be the β-glucopyranoside of selaginellol (**1**). The HMBC correlation from H-1″ to C-4′ indicated that the β-glucopyranosyl part was located at C-4′. According to our previously acidic hydrolysis of rel-(7*R*,8*S*)-3,3′,5-trimethoxy-4′,7-epoxy-8,5′-neoligna-4,9,9′-triol 4-*O*-β-d-glucopyranoside (**11**) [8], the sugar in the plant is d-glucose. The absolute configuration of the aglycone was elucidated to be the same as that of selaginellol (**1**) by comparison of its ECD spectrum [Δ*ε* −0.37 (273)] with that of **1**. Therefore, compound **2** is selaginellol 4′-*O*-β-d-glucopyranoside.

The known compounds were determined as (−)-syringaresinol (**3**) [10], (−)-lariciresinol (**4**) [11], 7*S*,7′*S*,8*R*,8′*R*-icariol A_2_ (**5**) [12], lyoniside (**6**) [13], (−)-8,8′-bisdihydrosiringenin (**7**) [14], (7*S*,8*R*)-3,3′,5-trimethoxy-4′,7-epoxy-8,5′-neoligna-4,9,9′-triol (**8**) [8], and dihydrosinapyl alcohol (**9**) [15], by comparing their NMR data (for all known compounds) and optical rotation values (for the neolignans) with those reported in the literature.

All of these compounds (**1**–**9**), along with those previously isolated from the plant, including (7*S*,8*R*)-4,9-dihydroxy-3,3′,5-trimethoxy-4′,7-epoxy-8,5′-neolignan-9′-oic acid methyl ester (**10**) [8], rel-(7*R*,8*S*)-3,3′,5-trimethoxy-4′,7-epoxy-8,5′-neoligna-4,9,9′-triol 4-*O*-β-d-glucopyranoside (**11**) [8], rel-(7*R*,8*S*)-3,3′,5-trimethoxy-4′,7-epoxy-8,5′-neoligna-4,9,9′-triol 9-*O*-β-d-glucopyranoside (**12**) [8], 3,3′,5-trimethoxy-4′,7-epoxy-8,5′-neolign-7-ene-4,9,9′-triol 9-*O*-β-d-glucopyranoside (**13**) [8], 5-carboxymethyl-7,4′-dihydroxyflavanone 7-*O*-β-d-glucopyranoside (**14**) [7], *N*-selaginelloyl-l-phenylalanine (**15**) [5], paucine 3′-*O*-β-d-glucopyranoside (**16**) [8], paucine (**17**) [8], and *N* ^1^-*cis*-*p*-coumaroylagmatine (**18**) [8], were evaluated for the inhibitory activity against platelet aggregation induced by ADP or collagen. As shown in Table 2, compounds **8**, **11**, **12**, **14**, and **16** showed potential inhibitory activity against ADP-induced platelet aggregation with IC_50_ values of 80.84, 35.76, 42.47, 27.70, and 59.19 µM, respectively, as compared with the positive control tirofiban (IC_50_ = 25.32 µM). Compounds **8**, **11**, **12**, and **14** also showed the activity against collagen-induced platelet aggregation with IC_50_ values of 146.70, 31.17, 24.57, and 26.25 µM, respectively, as compared with the positive control tirofiban (IC_50_ = 148.20 µM). The dihydrobenzofuran neolignans (**8**, **11**, and **12**) are more potent than the benzofuran neolignan (**13**) and other types of neolignans (**1**–**7**). Glucosidation of the dihydrobenzofuran neolignans (**11** and **12**) is helpful for the activity as compared the bioassay result of **11** and **12** with that of **8**.

**Table 2:** The effect of compounds on rabbit platelet aggregation induced by ADP (10 μM) or collagen (2.5 μg/mL)

Compound	ADP (IC_50_ µM)	Collagen (IC_50_ µM)
**8**	80.84	146.70
**11**	35.76	31.17
**12**	42.47	24.57
**14**	27.70	26.25
**16**	59.19	>200
Tirofiban (positive control)	25.32	148.20

## Experimental Section

### General Experimental Procedures

Optical rotations were recorded using a JASCO P-1020 Polarimeter (Jasco Corp., Tokyo, Japan). UV spectra were taken on a Shimadzu UV-2401 PC spectrophotometer (Shimadzu, Kyoto, Japan). ECD spectra were recorded on a Chirascan CD spectrometer (Applied Photophysics Ltd., Leatherhead, UK). IR spectra were measured on a Bruker Tensor 27 FTIR Spectrometer (Bruker Corp., Ettlingen, Germany) with KBr disks. ^1^H and ^13^C NMR spectra were collected on Bruker Avance 400, DRX-500 or Avance III-600 spectrometers (Bruker Bio-Spin GmbH, Rheinstetten, Germany) with TMS as an internal standard. ESIMS and HRESIMS analyses were carried out on an API QSTAR Pulsar 1 spectrometer (Applied Biosystems/MDS Sciex, Foster City, CA, USA). HREIMS were carried out on a Waters AutoSpec Premier p776 spectrometer (Waters, Millford, MA, USA). Silica gel G (80–100 and 300–400 mesh, Qingdao Meigao Chemical Co., Ltd., Qingdao, China), C_18_ silica gel (40–75 μm, Fuji Silysia Chemical Ltd., Aichi, Japan), Sephadex LH-20 (GE Healthcare Bio-Sciences AB, Uppsala, Sweden), and D_101_ macroporous resin (Qingdao Marine Chemical Ltd., Qingdao, China) were used for column chromatography, and silica gel GF_254_ (Qingdao Meigao Chemical Co., Ltd.) was used for preparative TLC as precoated plates. TLC spots were visualized under UV light at 254 nm and by dipping into 5 % H_2_SO_4_ in alcohol followed by heating. Semipreparative HPLC was performed on an Agilent 1200 series pump (Agilent Technologies, Santa Clara, USA) equipped with a diode array detector and an Agilent Zorbax SB-C_18_ column (5.0 μm, *ϕ* 9.4 × 250 mm).

### Plant Material

The whole plant of *S.* *moellendorffii* was collected from Jingxi County of Guangxi Zhuang Autonomous Region in 2008. A voucher specimen (No. JX0801) was identified by one of the authors (Chun-Lin Long) and deposited at the Key Laboratory of Economic Plants and Biotechnology, Kunming Institute of Botany, Chinese Academy of Sciences.

### Extraction and Isolation

The air-dried, powdered *S. moellendorffii* plants (15 kg) were exhaustively extracted with MeOH (45 L × 3) at 60 °C. The solvent was removed to give a residue (0.89 kg). The crude extract was subjected to chromatography on a D_101_ macroporous resin column eluted successively with H_2_O, 35 % EtOH, and 95 % EtOH to give three portions (I–III), respectively. Portion II (398 g) was subjected to column chromatography (silica gel G; CHCl_3_/MeOH, 1:0 → 0:1, v/v) to yield six fractions (A–F). Fr. A was subjected to column chromatography (silica gel G; petroleum ether/EtOAc, 15:1 → 0:1, v/v) to yield four fractions (A1–A4). Fr. A1 was purified by column chromatography (silica gel G; CHCl_3_-acetone, 15:1, v/v) to obtain **9**. Fr. A2 was chromatographed on a Sephadex LH–20 column (MeOH) to give subfractions A2–1 and A2–2. Subfraction A2–1 was subjected to chromatography on a silica gel G column (CHCl_3_-acetone, 20:1, v/v) and then further purified by semi-preparative HPLC (MeCN/H_2_O, 30:70, v/v) to yield **4** (14.9 mg, *t*_R_ = 13.099 min). Subfraction A2-2 was purified by preparative TLC (CHCl_3_/MeOH, 10:1, v/v) to obtain **3** (24.6 mg). Fr. A3 was chromatographed over a C_18_ silica gel column (MeOH/H_2_O, 30:70, v/v), a Sephadex LH-20 column (MeOH), and a silica gel G column (CHCl_3_/MeOH/H_2_O, 50:1:0.25), and purified by semi-preparative HPLC (MeOH/H_2_O, 40:60, v/v) to obtain **5** (15.5 mg, *t*_R_ = 5.864 min). Fr. A4 was chromatographed on a Sephadex LH-20 column (MeOH), a C_18_ silica gel (MeOH/H_2_O, 50:50, v/v), and a silica gel G column (CHCl_3_/MeOH, 60:1, v/v), and purified by semi-preparative HPLC (MeCN/H_2_O, 30:70, v/v) to yield **7** (2.0 mg, *t*_R_ = 7.716 min), **8** (2.0 mg, *t*_R_ = 8.917 min) and **1** (4.0 mg, *t*_R_ = 13.652 min). Fr. D was chromatographed on a C_18_ silica gel column (MeOH/H_2_O, 30:70, v/v), a Sephadex LH-20 column (MeOH), and a silica gel G column (CHCl_3_/MeOH/H_2_O, 100:10:0.5, v/v), and purified by semi-preparative HPLC (MeOH/H_2_O, 40:60, v/v) to yield **2** (7.8 mg, *t*_R_ = 12.138 min) and **6** (4.4 mg, *t*_R_ = 18.734 min).

#### Selaginellol (**1**)

Pale yellow oil (MeOH); [α]_D_^24^ −50.4 (*c* 0.40, MeOH); UV (CH_3_OH) *λ*_max_ (log *ε*) 280 (3.31), 228 (3.98) nm; ECD Δ*ε* (*c* 0.010, MeOH) −0.12 (273), −3.84 (214), +3.63 (197); IR (KBr) *v*_max_ 3428, 1614, 1518, 1496, 1461, 1431, 1289, 1217, 1114 cm^−1^; ^1^H and ^13^C NMR data, see Table 1; positive ion ESIMS *m/z* 415 [M+Na]^+^; positive ion HRESIMS *m/z* 415.1729 [M+Na]^+^ (calcd for C_21_H_28_O_7_Na^+^, 415.1727).

#### Selaginellol 4′-*O*-β-d-glucopyranoside (**2**)

Pale yellow solid (MeOH); [α]_D_^22^ −57.8 (*c* 0.26,MeOH); UV (CH_3_OH) *λ*_max_ (log *ε*) 274 (3.80) nm; ECD Δ*ε* (*c* 0.011, MeOH) −0.37 (273), −6.17 (210), +3.20 (200); IR (KBr) *v*_max_ 3425, 1615, 1518, 1461, 1428, 1325, 1216, 1113, 1071 cm^−1^; ^1^H and ^13^C NMR data, see Table 1; positive ion ESIMS *m/z* 577 [M+Na]^+^; HREIMS *m/z* 554.2365 [M]^+^ (calcd for C_27_H_38_O_12_, 554.2363).

### In Vitro Platelet Aggregation Assay

In vitro platelet aggregation was conducted using the turbidimetric method with a minor modification [16, 17]. Briefly, blood was withdrawn from the carotid artery of New Zealand rabbits,and anticoagulated with 3.8 % sodium citrate (1:9 citrate/blood, v/v) and centrifuged for 15 min at 950 rpm to prepare platelet-rich plasma (PRP) or 10 min at 3000 rpm to obtain platelet-poor plasma (PPP). The platelet concentration was adjusted to 3 × 10^8^ platelets/mL. PRP in 270 μL was preincubated at 37 °C for 5 min in the cuvette with 20 μL of sample or vehicle (saline), and then platelet aggregation was induced by 10 μL ADP (10 μM) or collagen (2.5 μg/mL). The maximum platelet aggregation rate was determined within 5 min with continuous stirring at 37 °C using four-channel aggregometer (Beijing Steellex Science Instrument Company, China).

For each compound, five concentrations were chosen and a percentage inhibition-concentration curve was derived. From this curve the IC_50_ value was calculated as the concentration of inhibitor causing a 50 % inhibition of the aggregation using SPSS software.

## Electronic supplementary material

Below is the link to the electronic supplementary material.

Supplementary material 1 (PDF 782 kb)
