# Supplementary material for: Neolignans from Selaginella moellendorffii
Source: Nat Prod Bioprospect. 2016 Apr 7;6(3):161–6. doi: 10.1007/s13659-016-0095-5 (PMC5385659; doi:10.1007/s13659-016-0095-5)

Supplementary Material for

Neolignans from *Selaginella moellendorffii*

Jing-Xian Zhuo · Yue-Hu Wang · Xing-Li Su · Ren-Qiang Mei · Jun Yang · Yi Kong · Chun-Lin Long

Jing-Xian Zhuo and Yue-Hu Wang contributed equally to this work.

J.-X. Zhuo · Y.-H. Wang · R.-Q. Mei ·J. Yang
Key Laboratory of Economic Plants and Biotechnology, and Yunnan Key Laboratory for Wild Plant Resources, Kunming Institute of Botany, Chinese Academy of Sciences, Kunming 650201, China

C.-L. Long (✉)
Key Laboratory of Economic Plants and Biotechnology, and Yunnan Key Laboratory for Wild Plant Resources, Kunming Institute of Botany, Chinese Academy of Sciences, Kunming 650201, China, or College of Life and Environmental Sciences, Minzu University of China, Beijing 100081, China
e-mail: long@mail.kib.ac.cn

X.-L. Su · Y. Kong (✉)
School of Life Science & Technology, China Pharmaceutical University, Nanjing 210009, China
e-mail: yikong668@163.com

Contents

**Figure S1.** ^{1}H NMR spectrum of **1** (CD_3OD, 600 MHz).
**Figure S2.** ^{13}C NMR spectrum of **1** (CD_3OD, 150 MHz).
**Figure S3.** HSQC spectrum of **1**.
**Figure S4.** ^{1}H–^{1}H COSY spectrum of **1**.
**Figure S5.** HMBC spectrum of **1**.
**Figure S6.** ROESY spectrum of **1**.
**Figure S7.** HRESIMS spectrum of **1.**
**Figure S8.** ^{1}H NMR spectrum of **2** (CD_3OD, 600 MHz).
**Figure S9.** ^{13}C NMR spectrum of **2** (CD_3OD, 150 MHz).
**Figure S10.** HSQC spectrum of **2**.
**Figure S11.** ^{1}H–^{1}H COSY spectrum of **2**.
**Figure S12.** HMBC spectrum of **2**.
**Figure S13.** ROESY spectrum of **2**.
**Figure S14.** HREIMS spectrum of **2.**

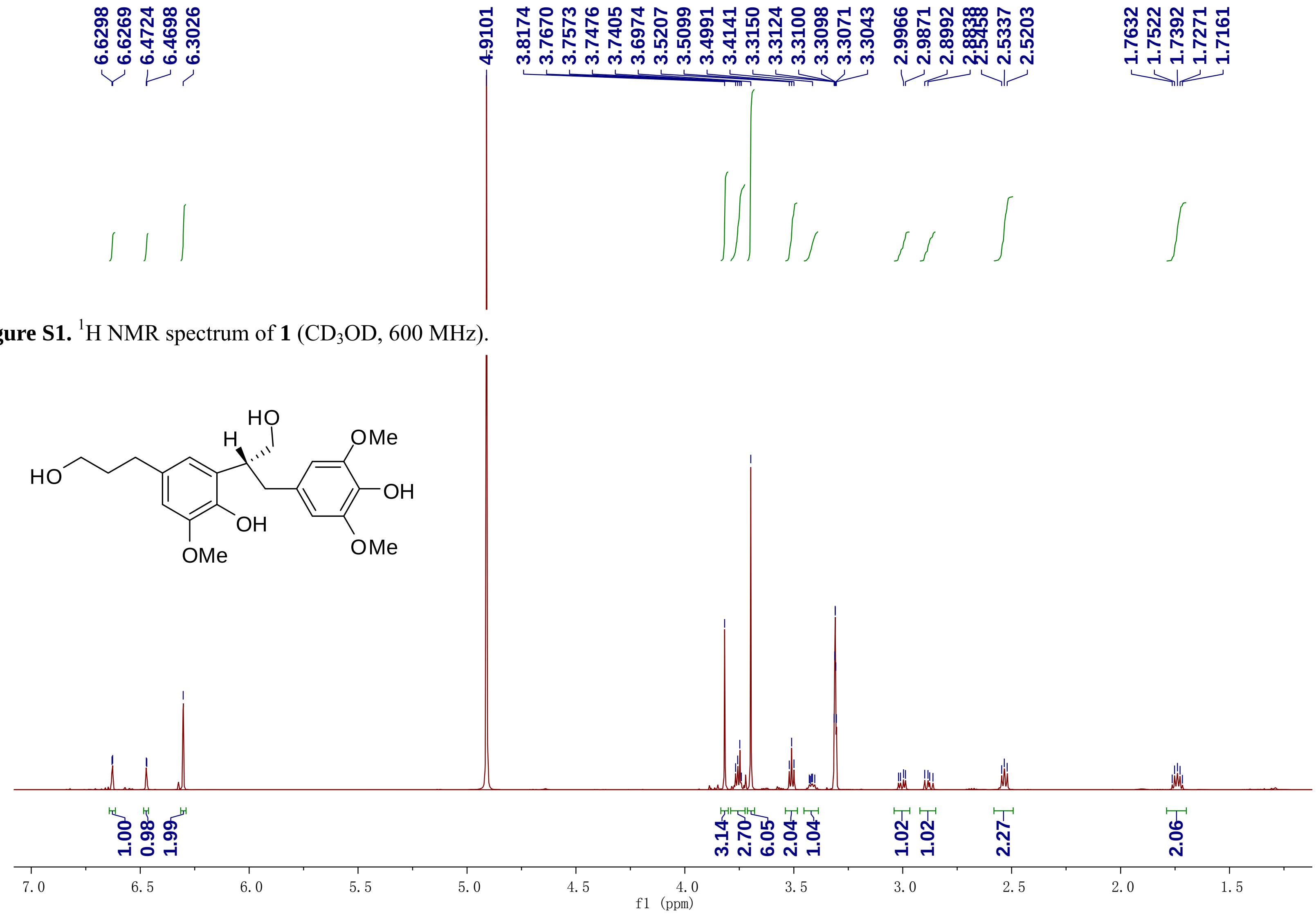

Figure S1. ^{1}H NMR spectrum of **1** (CD_3OD, 600 MHz).

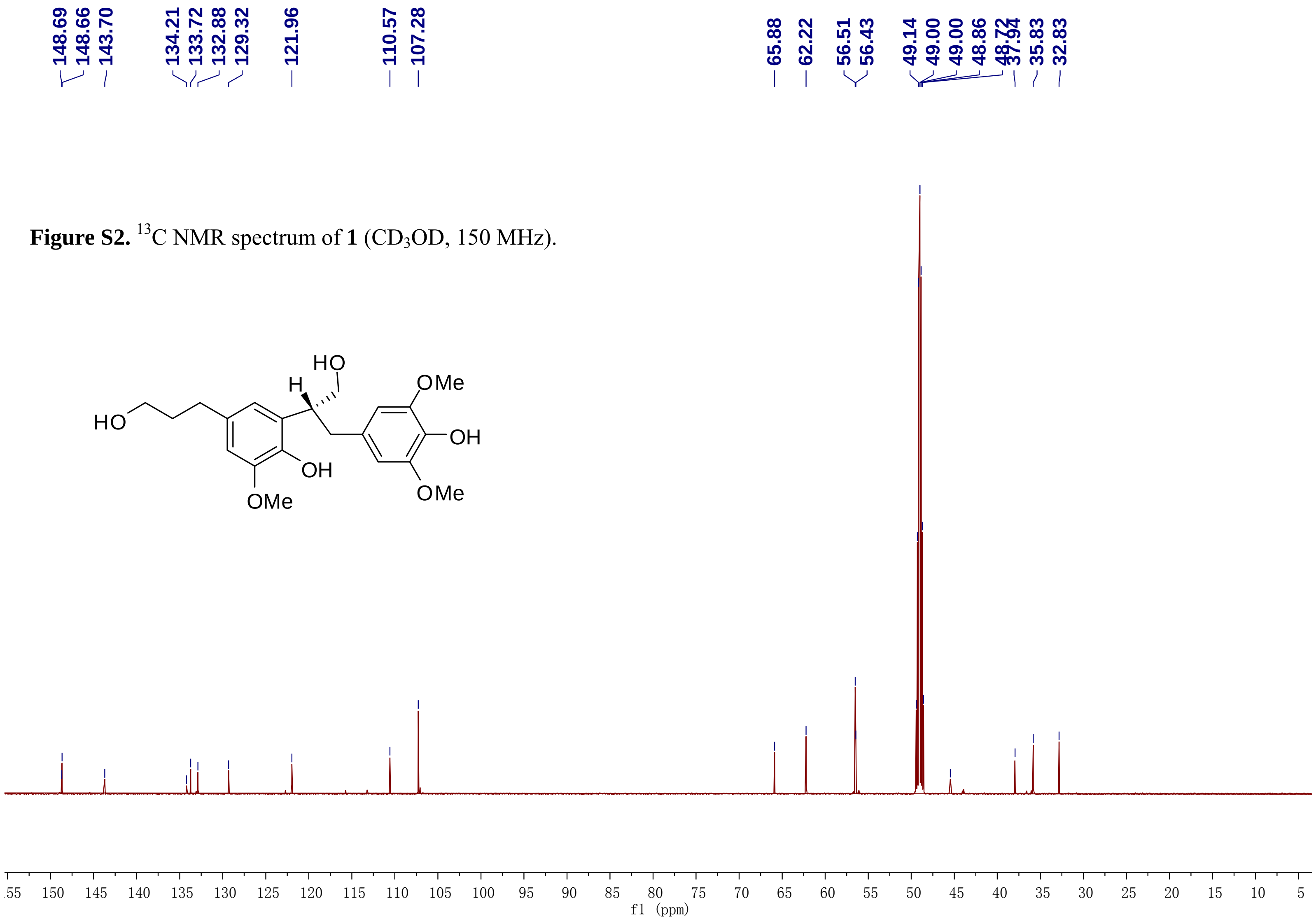

Figure S2. ^{13}C NMR spectrum of **1** (CD_3OD, 150 MHz).

Figure S3. HSQC spectrum of **1**.

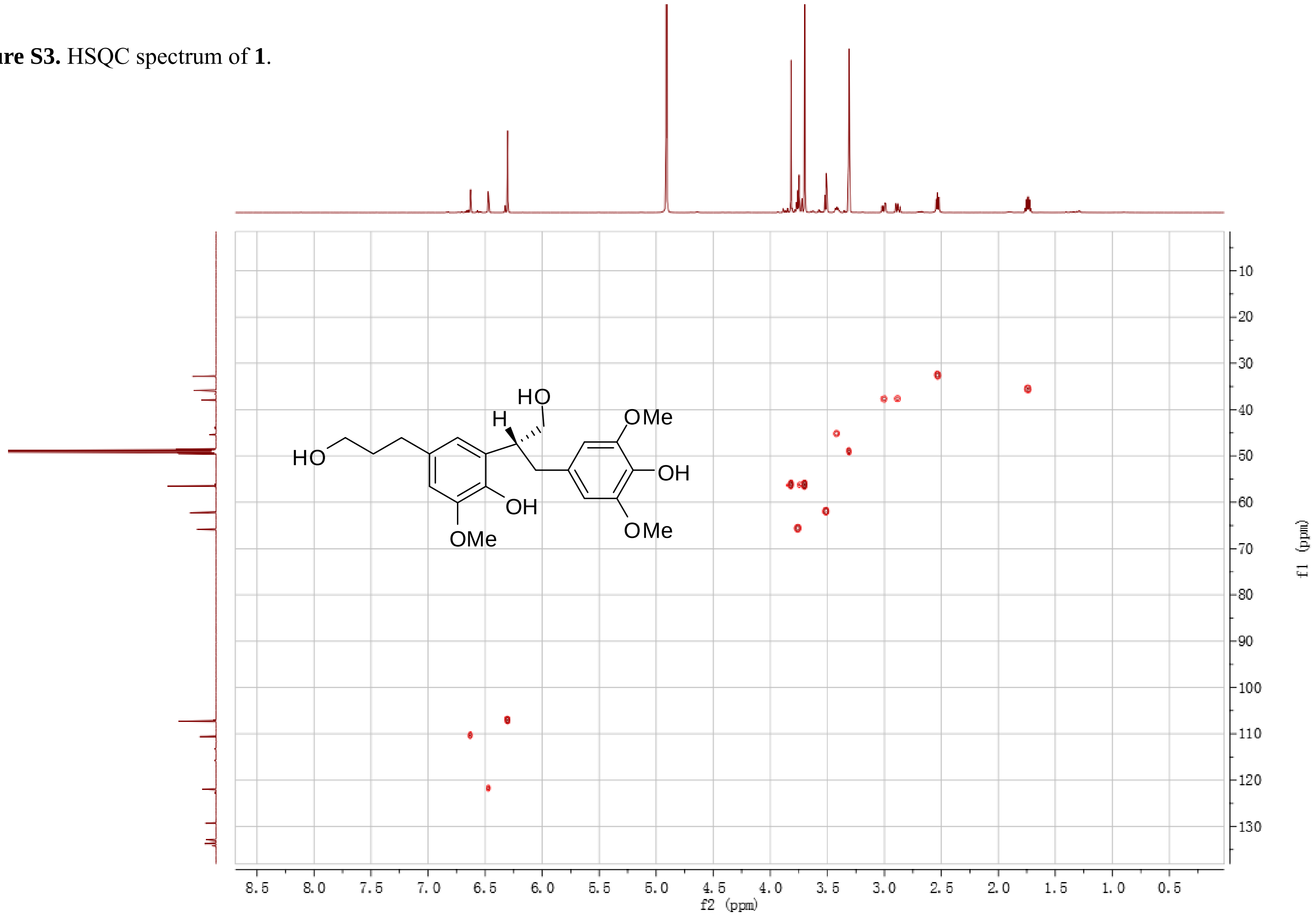

Figure S4. ${}^{1}H$–${}^{1}H$ COSY spectrum of **1**.

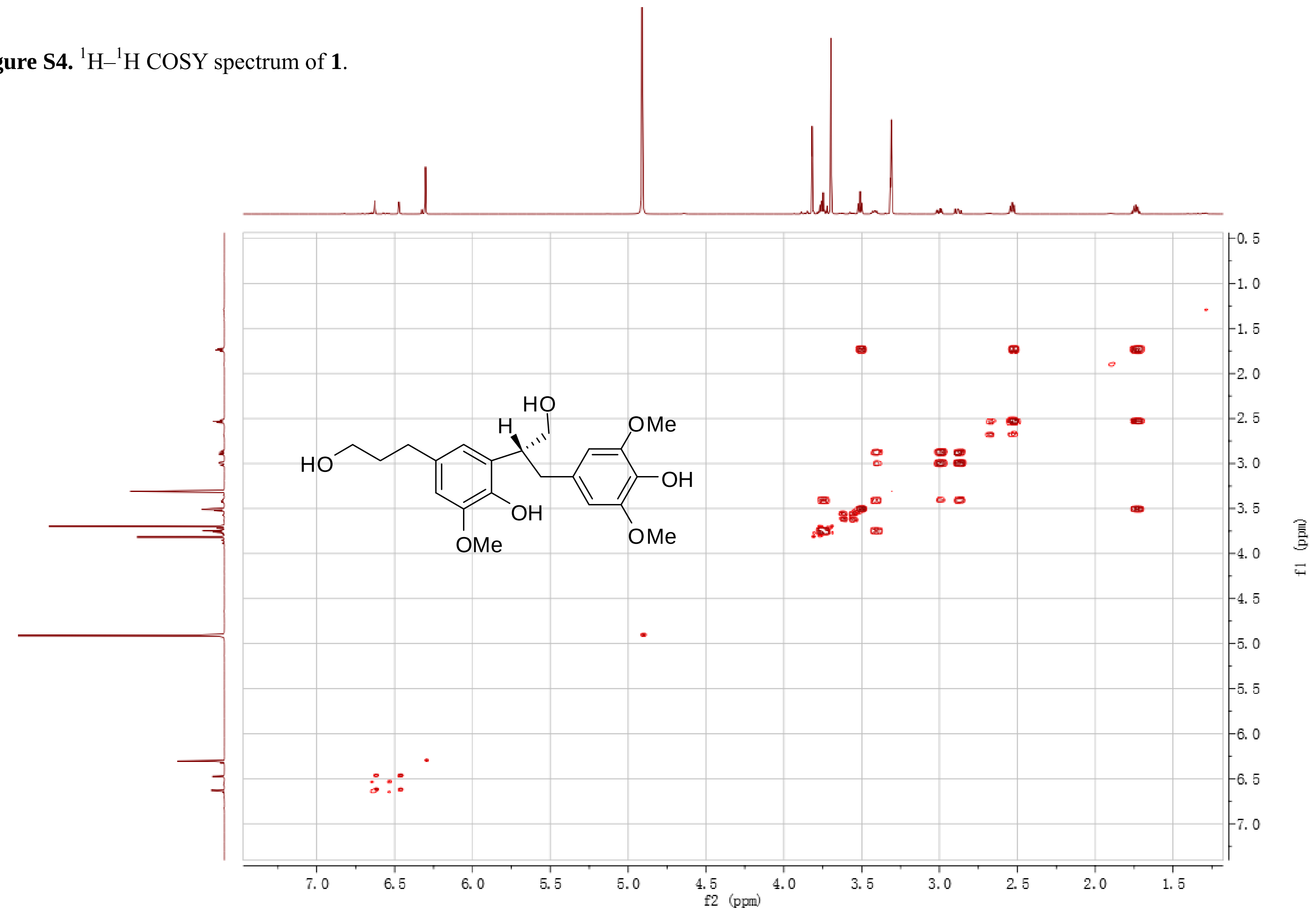

Figure S5. HMBC spectrum of **1**.

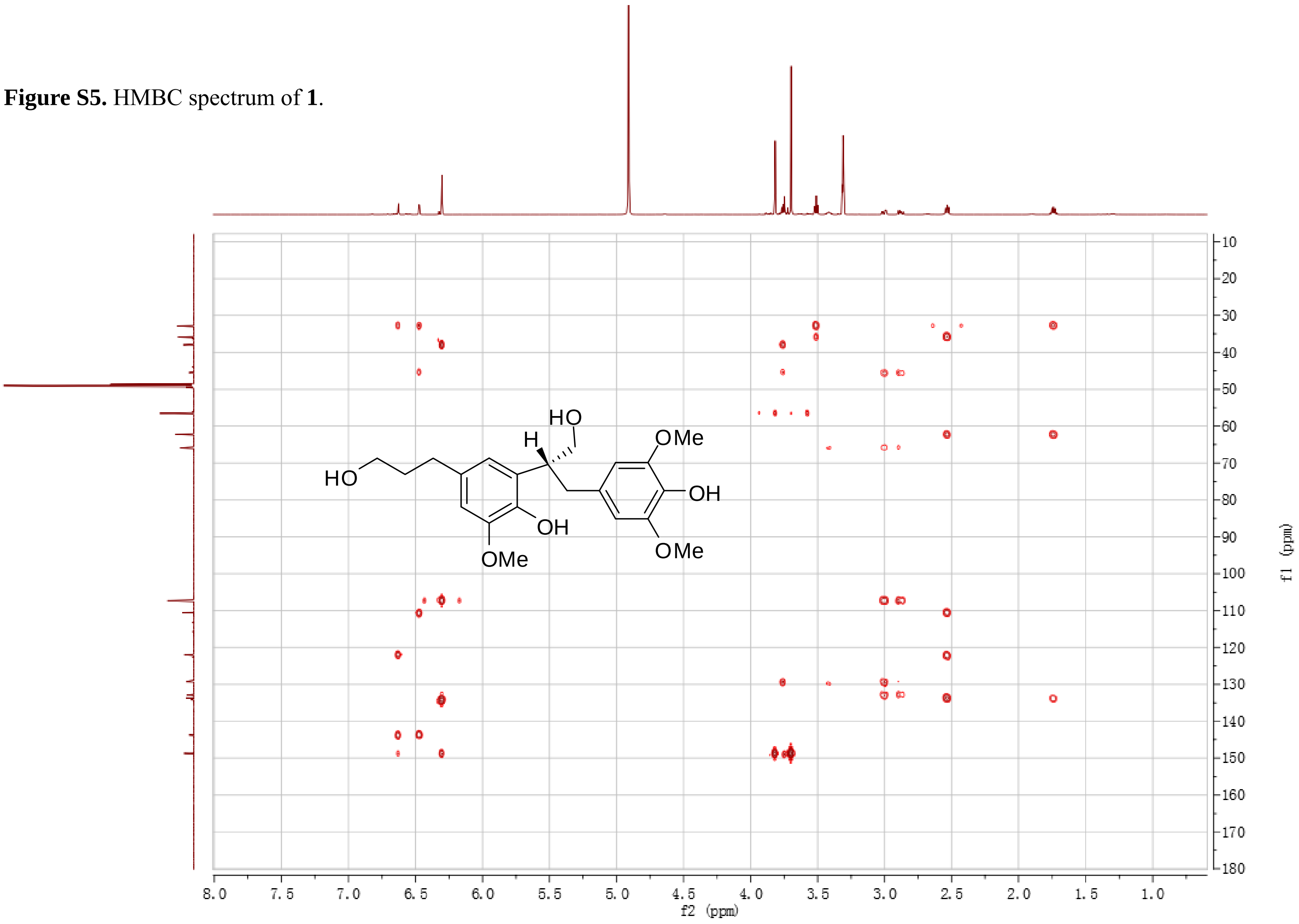

Figure S6. ROESY spectrum of **1**.

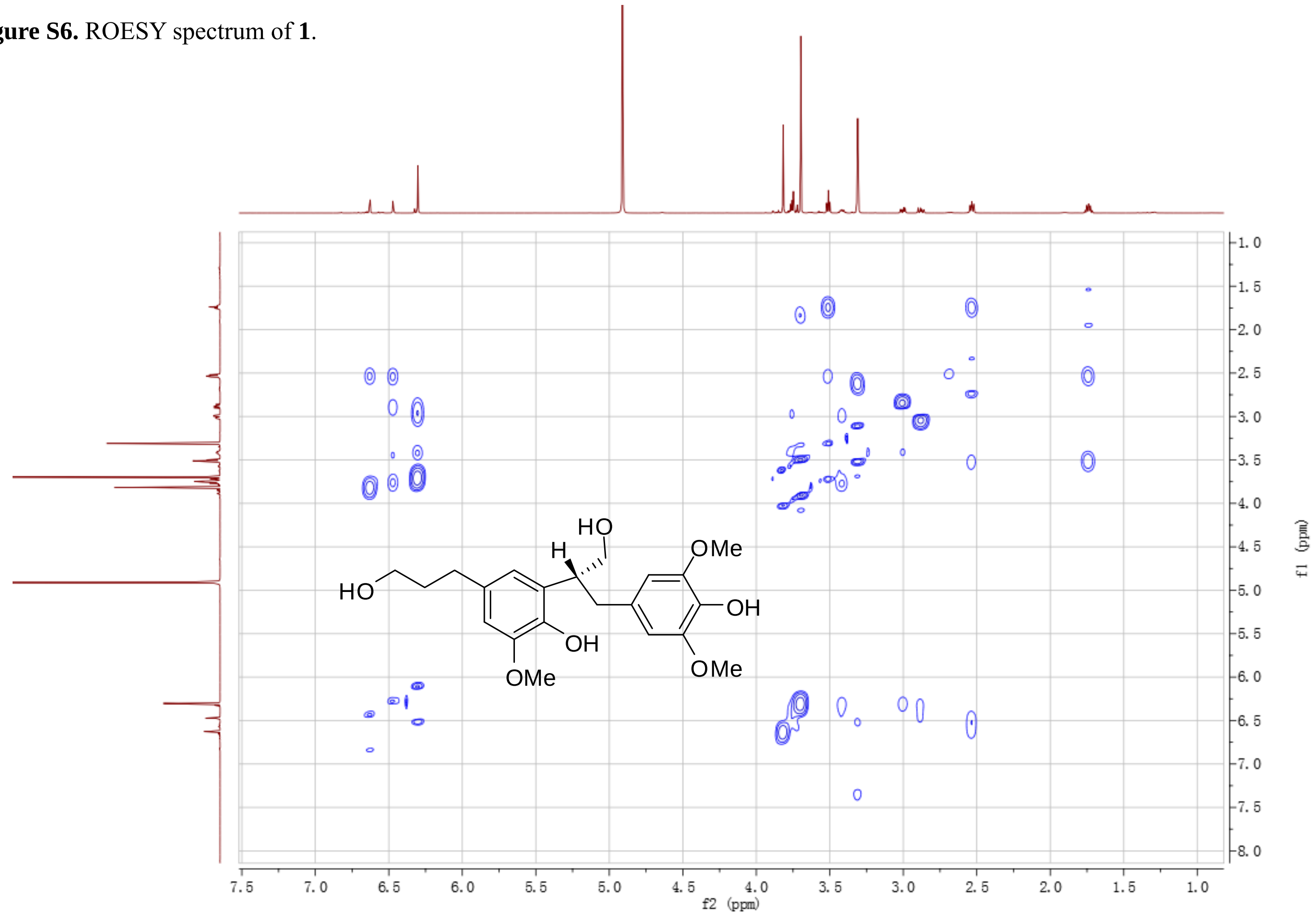

Figure S7. HRESIMS spectrum of **1**.

Qualitative Analysis Report

**Data Filename**	psm95.d	**Sample Name**	psm95
**Sample Type**	Sample	**Position**	P1-D7
**Instrument Name**	Instrument 1	**User Name**	
**Acq Method**	SIBU.m	**Acquired Time**	4/8/2015 12:38:08 PM
**IRM Calibration Status**	Success	**DA Method**	Default.m
**Comment**			

**Sample Group**		**Info.**
**Acquisition SW Version**	6200 series TOF/6500 series Q-TOF B.05.01 (B5125.2)	

User Spectra

Fragmentor Voltage	Collision Energy	Ionization Mode
135	0	ESI

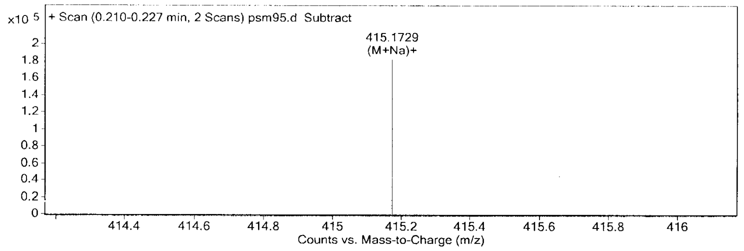

Peak List

m/z	z	Abund	Formula	Ion
323.1756	1	334075.91		
324.1789	1	72398.36		
393.1907	1	40879.24		
410.2174	1	50268.82		
415.1729	1	186347.03	C21 H28 O7	(M+Na)+
416.1761	1	39789.51	C21 H28 O7	(M+Na)+
431.1467	1	175852.97		
432.1499	1	39748		
645.3435	1	134004.41		
646.3465	1	58985.05		
715.3587	1	31837.56		
807.356	1	40185.57		

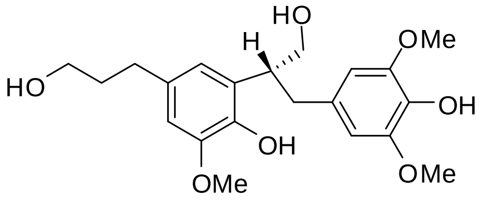

Formula Calculator Element Limits

Element	Min	Max
C	3	100
H	0	200
O	0	60

Formula Calculator Results

Formula	CalculatedMass	CalculatedMz	Mz	Diff. (mDa)	Diff. (ppm)	DBE
C21 H28 O7	392.1835	415.1727	415.1729	-0.2	-0.4	8.0000

--- End Of Report ---

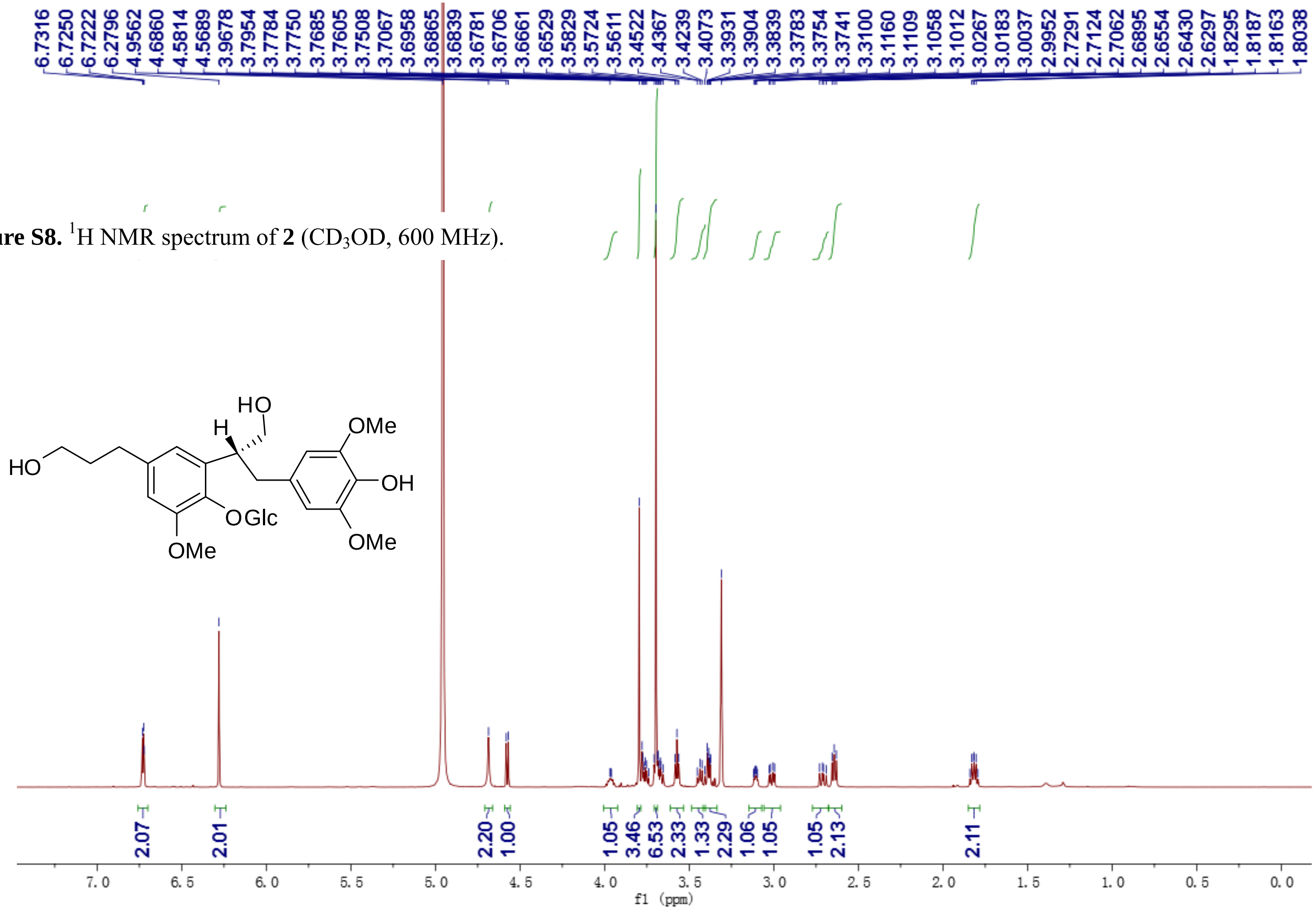

Figure S8. ^{1}H NMR spectrum of **2** (CD_3OD, 600 MHz).

Figure S9. ^{13}C NMR spectrum of **2** (CD_3OD, 150 MHz).

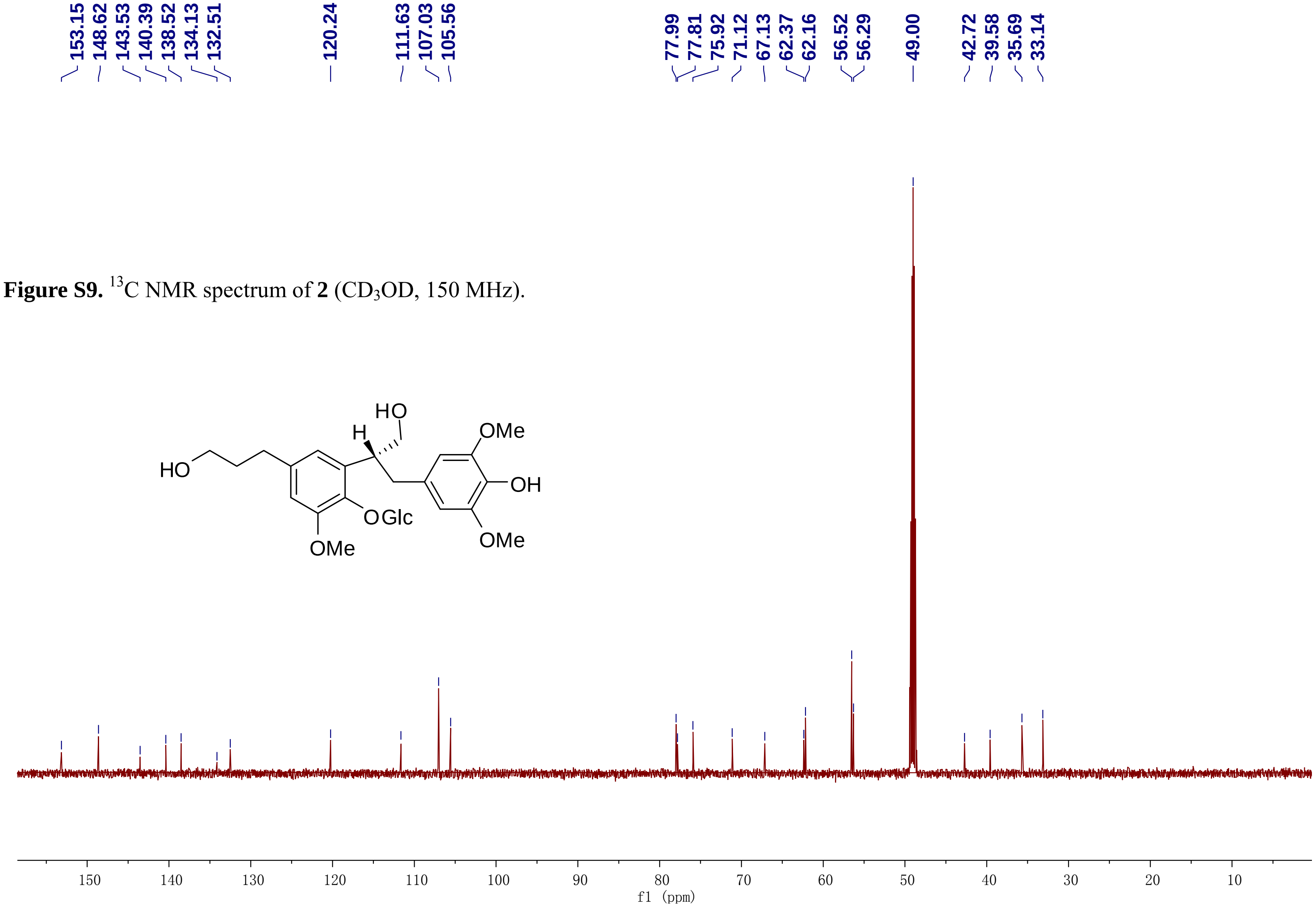

Figure S10. HSQC spectrum of **2**.

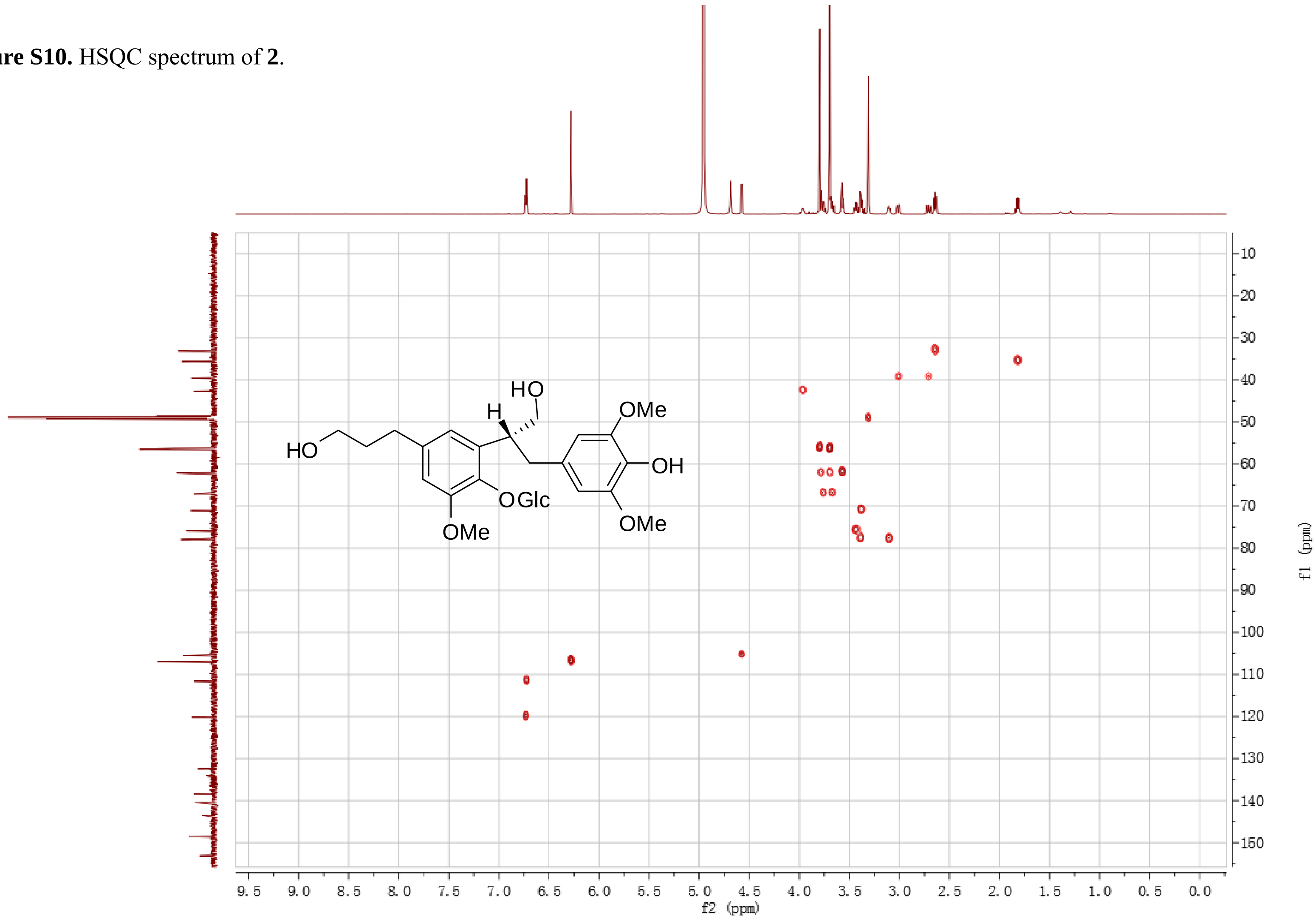

Figure S11. ^{1}H–^{1}H COSY spectrum of **2**.

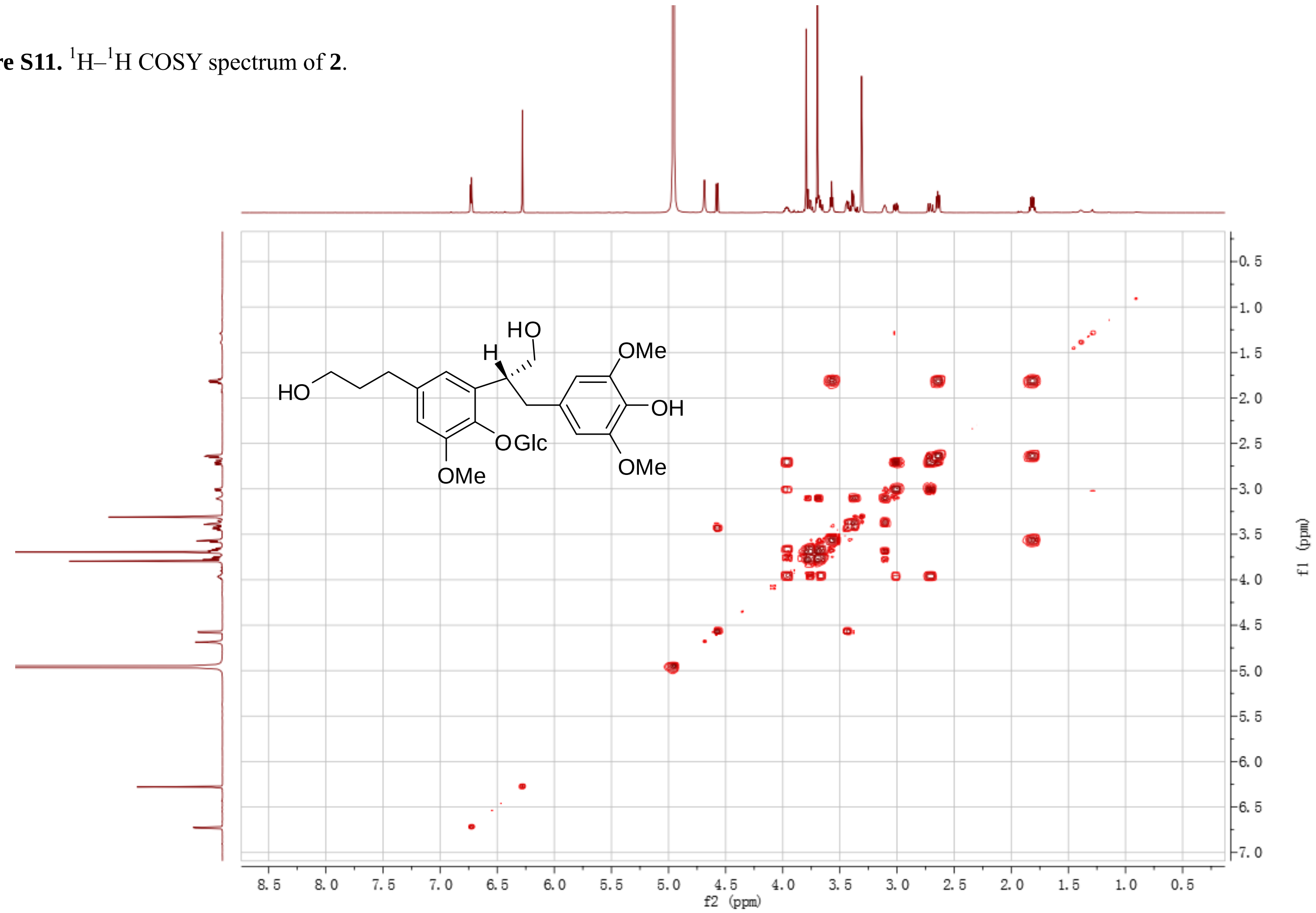

Figure S12. HMBC spectrum of **2**.

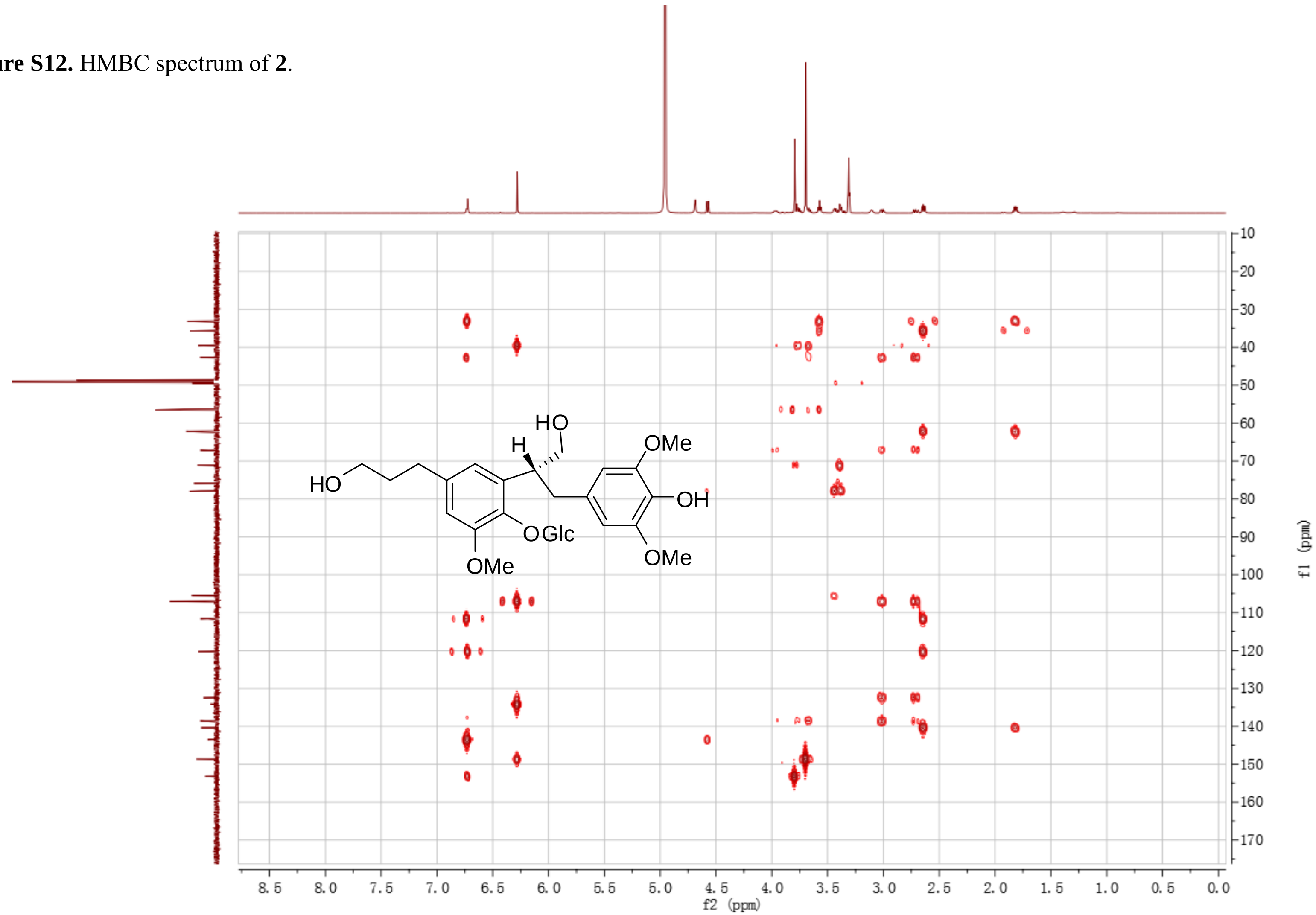

Figure S13. ROESY spectrum of **2**.

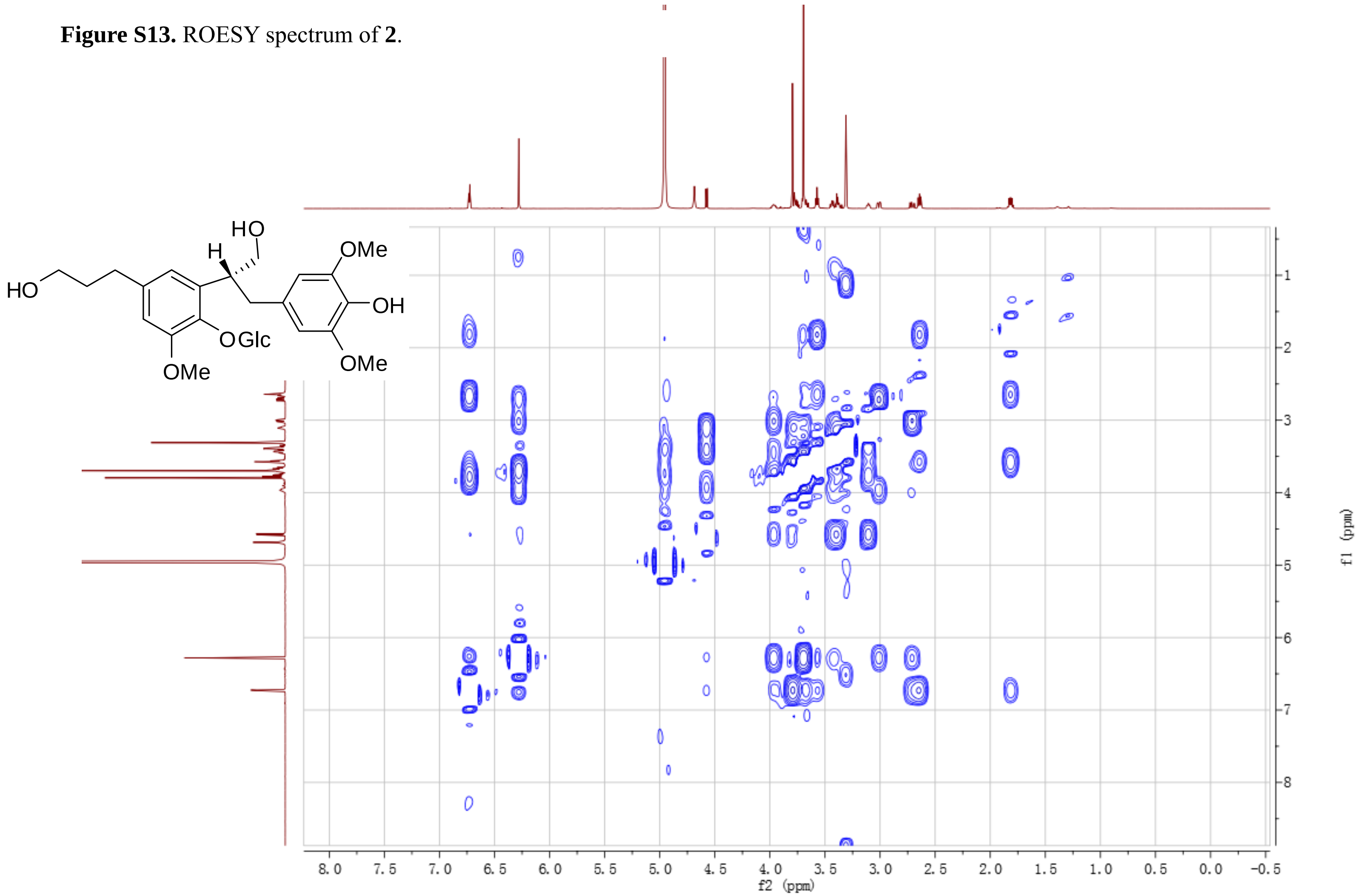

Figure S14. HREIMS spectrum of **2**.

Elemental Composition Report

Page 1

Single Mass Analysis
Tolerance = 10.0 PPM / DBE: min = -10.0, max = 120.0
Selected filters: None

Monoisotopic Mass, Odd and Even Electron Ions
20 formula(e) evaluated with 1 results within limits (up to 51 closest results for each mass)
Elements Used:
C: 0-200 H: 0-400 O: 11-13
psm80
15:27:54 29-Oct-2014
Voltage EI+

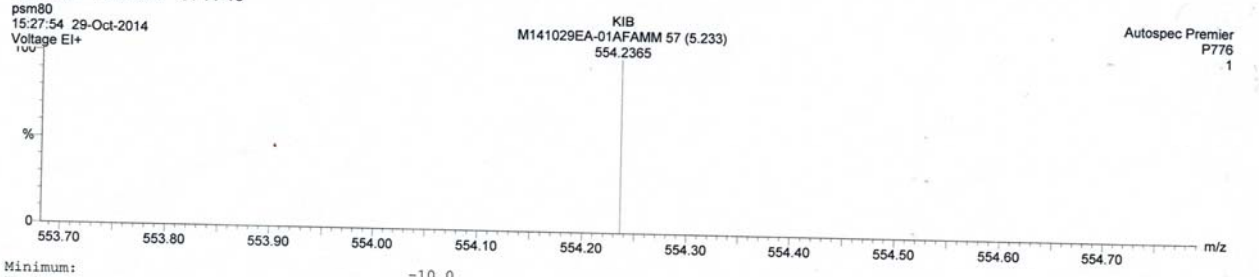

Mass	Calc. Mass	mDa	PPM	DBE	i-FIT	Formula
Minimum:				-10.0		
Maximum:		200.0	10.0	120.0		
554.2365	554.2363	0.2	0.4	9.0	5546026.0	C27 H38 O12

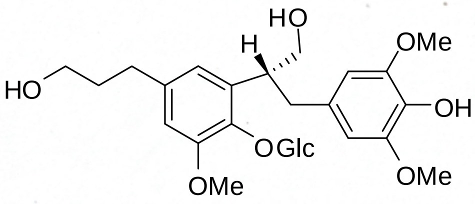

Supplement: Supplementary file 1 — Supplementary material 1 (PDF 782 kb) [file 13659_2016_95_MOESM1_ESM.pdf]
